# Modified Atmosphere and Humidity Film Reduces Browning Susceptibility of Oriental Melon Suture Tissue during Cold Storage

**DOI:** 10.3390/foods9091329

**Published:** 2020-09-21

**Authors:** Me-Hea Park, Eun-Ha Chang, Hae-Jo Yang, Jung-Soo Lee, Gyung-Ran Do, Hyun Jong Song, Min-Sun Chang, Kang-Mo Ku

**Affiliations:** 1 Postharvest Research Division, National Institute of Horticultural & Herbal Science, Wanju 55365, Korea; xsede@naver.com (E.-H.C.); gowh1231@korea.kr (H.-J.Y.); lljs808@korea.kr (J.-S.L.); aeru@korea.kr (M.-S.C.); 2 Planning and Coordination Division, National Institute of Horticultural & Herbal Science, Wanju 55365, Korea; microdo@korea.kr; 3 Department of Horticulture, College of Agriculture and Life Sciences, Chonnam National University, Gwangju 61186, Korea; 183247@jnu.ac.kr; 4 Department of Interdisciplinary Smart Agriculture at Chonnam National University, Gwangju 61186, Korea

**Keywords:** browning, modified atmosphere packaging, moisture loss, oriental melon, relative humidity

## Abstract

Oriental melons have a relatively short shelf life as they are harvested during the summer season and susceptible to cold-induced injuries. Typical chilling injury when stored at 4 °C is expressed as browning of the fruit suture. To prolong the shelf life and reduce browning of the fruit, the effects of modified atmosphere packaging (MAP), X-tend modified atmosphere (MA)/modified humidity (MH) bulk packaging (XF), and polyethylene (PE) packaging, on oriental melons were investigated during storage at 4 °C and 10 °C for 14 days and under retail display conditions at 20 °C. The O_2_ concentrations in PE packages stored at 4 °C and 10 °C ranged from 17.4 to 18.5%, whereas those in XF packages were reduced to 16.3–16.6%. The CO_2_ content of XF package (4.2–4.6%) was higher than that of PE package (1.4–1.9%) stored at 4 °C or 10 °C. Relative humidity (RH) saturated in the PE packages but not in the XF packages after seven days of storage. Furthermore, PE packages performed better at maintaining melon weight and firmness than XF packages during storage at 10 °C for 14 days and under retail display conditions at 20 °C. PE and XF packages effectively reduced the browning index of the peel and white linear sutures of oriental melons compared with the unpackaged control during cold storage at 4 °C, and this observation was maintained at the retail display condition at 20 °C. The enhanced CO_2_ levels, reduced O_2_ levels, and optimal RH values that were provided by the MAP, prevented the browning symptoms, and improved the marketability and shelf life of oriental melons.

## 1. Introduction

The oriental melon (*Cucumis melo* var L.) is an important agricultural commodity and famous summer fruit in Korea. The oriental melon has light yellow smooth skin and white flesh, with a white suture between the yellow skin, holding a completely different appearance and taste compared with other melons including honeydew and cantaloupe [1]. The oriental melon has high sugar content, calcium, and vitamin C [2]. It is commonly cultivated directly in the open field, in the middle of April, or planted after growing seedlings, in the end of May. The melons are normally harvested from May to August, which is the rainy season with high temperatures. Therefore, it is difficult to maintain the quality of the oriental melon at room temperature, during storage, and shipping. The *Cucumis melo* var L *makuwa* oriental melon has a shelf life after harvest of only ~10 days at room temperature due to its typical climacteric behavior and thin pericarp [3]. The oriental melon quality during storage at room temperature (23 °C) is affected by softening, senescence, browning, and overall decay of the fruit [1,4,5]. Due to its unique appearance and taste, oriental melons are exported from Korea to other countries [6]; therefore, low temperature storage strategies are necessary to extend their shelf life. However, under low temperature storage, oriental melons can develop cold injuries (CIs), such as soaking and *Alternaria* rot [7]. Browning of the peel and “suture” are the main factors that lead to oriental melon postharvest loss. Peel browning increases with low melon storage temperatures [8], with the optimal oriental melon storage conditions being within 7–10 °C and high relative humidity (RH) of 90–95% [2]. Ethanol application has reduced the internal ethylene concentration of harvested oriental melons and maintained postharvest storage quality [1,9], whereas heat treatment at 38 °C for 48 h also prevented CIs outcome [7]. Furthermore, melon fruit senescence and decay can be controlled by methyl jasmonate [10], chitosan [11], and modified atmosphere packaging (MAP) [12].

MAP is a technology used to extend the shelf life of fruits and vegetables. Packaging with plastic films results in the creation of a modified atmosphere compared with the exterior environment, with higher CO_2_ and water vapor levels, and lower O_2_ levels, due to respiration and reduction of moisture loss from the commodity [13]. Reduced O_2_ or elevated CO_2_ levels inside the package can reduce ethylene production, delay ripening and softening, and slow various compositional changes associated with ripening. The use of MAP alleviates CIs in horticultural crops such as sweet corn [14] and sweet cherries [15]. X-tend films (StePac L.A., Tefen, Israel) were developed to modify the atmosphere and humidity inside the package and prolong the product quality; therefore, extending the shelf life of fresh products, such as melon, broccoli, green onions, mango, and honeysuckle fruits [16,17,18]. Porat et al. [19] demonstrated that the use of a MAP “bag-in-box” packaging with X-tend film reduces the incidence of rind disorder symptoms in citrus fruits. Moreover, X-tend films was developed to have higher permeability to water vapor by possessing microperforations, which will allow it to achieve enough in-pack relative humidity that will prevent the accumulation of condensed water on the produce [20]. This MAP and modified humidity packaging (MHP) was reported to effectively reduce CI symptoms in mangoes and tomatoes [21,22].

MAP in combination with low temperature storage is an effective way to improve the shelf life of crops. This study aimed to determine the effects of MAP using polyethylene (PE) film and MA/MH film on the quality attributes, CI, shelf life, and decay of oriental melons during storage at optimal (10 °C) and chilled temperature (4 °C) temperatures, and under retail display condition (20 °C).

## 2. Materials and Methods

### 2.1. Sample Preparation

Oriental melons (*Cucumis melo* var L. cv “Smart”) were harvested in August 2018 at optimum maturity from a plantation located in Sungju, South Korea, and used a day after harvest. For the experiment, fined whole fruit without defeat were selected and washed and dried to remove the extra water. Fruits were assessed for total soluble solids, color, firmness, elasticity, and weight before storage under different packaging conditions. For film processing, low-density polyethylene (PE) film (0.03 mm thickness; Tebangparteck, Yangju, Korea) and Xtend MA/MH bulk package (XF) with antifog (815-ST2, StePac, Israel) were used. The control melons were stored in a standard cardboard box without film treatment. The fruits were stored at 4 °C and 10 °C for 14 days and transferred to 20 °C for another five days to mimic the process of commercial melon distribution from producer to local market (temperature and relative humidity data throughout the experiment is available in Figure S1). After transferring to market display conditions (20 °C), the storage bags were opened. Physicochemical and sensory parameters were evaluated and compared among the three packaging groups that contained 12 fruits at the beginning of the experiment (Day 0).

### 2.2. In-Package Temperature, Humidity, and Headspace Gas Composition

The headspace gas composition (O_2_ and CO_2_ concentration) inside each package was monitored daily using a CheckMate 3 gas analyzer (PBI Dansensor, Ringsted, Denmark). In detail, first, the septum was attached on the packaging film at a placed with free space of packaging. To monitor the gas composition, the needle connecting to the analyzer pierced through the septum on the packing film. The needle was withdrawing when the measurement was finished. To monitor the temperature and humidity in the packages, data loggers (Watch dog, Spectrum Technology, Fort Worth, TX, USA) were placed inside the packages of each treatment and set to record temperature and RH every 30 min.

### 2.3. Weight Loss of Oriental Melon Fruit

Oriental melons were weighed at the beginning and at the end of the experiment. The weight loss (WL) percentage was calculated according to the following equation: WL(%) = ((IW − FW)/IW) × 100, in which the final weight (FW) was related to the initial weight (IW) of each sample.

### 2.4. Firmness Analysis

Firmness was measured at three points on the shoulder of each of 10 oriental melons from each group using a texture analyzer (TA Plus Lloyd Instruments, Ametek, Largo, FL, USA) connected to a computer, by applying a plunger of 5 mm in diameter. Texture analyzer was set up to Puncture methods which measured the hardness. In detail, the amount of force required to compress the radial pericarp surface of each oriental melon at a constant speed of 2.4 mm/s was recorded. The fruit firmness value was expressed as force per unit (N), firmness. The reported values represented the average value of 10 samples, with three measurements per sample, of each group.

### 2.5. Total Soluble Solids

The total soluble solid (TSS) content of the oriental melons was measured using a digital refractometer (PAL-1, Atago, Tokyo, Japan). Each whole oriental melon was cut in half, and each half was further divided into three parts. The juice from slices was extracted manually and put into the refractometer. The value of soluble solids content was expressed as Brix. The reported values represent the average value of 10 samples per group.

### 2.6. Surface Color Analysis

The surface color of each oriental melon was measured at three points on the peel with a reflectance colorimeter (Chroma Meter CR-400, Konica Minolta, Tokyo, Japan) using the Hunter color system. The color of each oriental melon was expressed as Hue value. The reported values represent the average of 12 samples per group. 

### 2.7. Determination of Browning Injury Index and Marketability

The browning of oriental melon peels and white linear sutures were measured in 15 individual fruits by an experienced investigator. The browning index assessment was performed using the following visual appearance scoring scale in relation to the portion of the fruit that was under investigation: 0, no symptoms; 1, 2–5% symptom; 2, 5–25% symptoms; 3, 25–50% symptoms; and 4, >50% symptoms. The browning index was determined using the following equation: (∑ (symptom scale × number of fruit at each scale))/(total number of fruit in the treatment).

Fruit marketability was assessed according to overall visual quality score: 5, excellent; 4, good; 3, fair; 2, bad; and 1, severe bad. The marketable limit was set as 3, and fruits with lower scores were considered unmarketable. Marketability data are presented as the percentage of marketable fruits that were affected within each treatment. The experiment was repeated thrice and the standard error of the mean for each parameter was calculated.

### 2.8. Light and Scanning Electron Microscopy for Tissue Structure Analysis

Tissue analysis was performed as previously described [23] with some modifications. Briefly, melon tissues were fixed in 2.5% glutaraldehyde (*v/v* in a 0.1 M phosphate buffer) at pH 7.2 in the presence of 4% sucrose (*w*/*v*) for 24 h. After three rinses (30 min, each) with the above indicated buffer, the specimens were post-fixed with 1% OsO_4_ *(w/v)* in the same buffer with 4% sucrose (*w*/*v*) for 4 h. They were then rinsed thrice (30 min, each) with the buffer, dehydrated in alcohol series, transferred to propylene oxide, and embedded in Epon epoxy resin. Semi-thin sections (2.5 µm) were prepared with an ultra-microtome and placed on glass slides. The Periodic Acid–Schiff (PAS) polysaccharide specific reaction was carried out, with tissues structures being shown in red color. Sections for staining were first plunged in 1% periodic acid (*w*/*v*) for 30 min, then in Schiffs reagent for 40 min, and in 5% sodium bisulfite (*w*/*v*) for 35 min. Sections were then rinsed in distilled water, dried on a warm plate, and mounted in Histomount. Negative control was performed by omitting the oxidation step with periodic acid. The samples were observed with a light microscope (Axioscop 2, Carl Zeiss, Jena, Germany). Cuticle thickness was measured with ImageJ. In order to examine the morphological characters, live tissues were examined on a SEM (SU-3500, Hitachi, Tokyo, Japan) operating at low vacuum mode [23]. 

### 2.9. Quantification and Composition of Epicuticular Wax

Oriental melon surface was peeled with a potato peeler; with a thickness of about 3–4 mm. Yellow peel and white suture tissue were separated with scissor. The surface area of each tissue was calculated by ImageJ. Two oriental melons were peeled for one biological replication. Chloroform (5 mL) was placed into a 20 mL glass vial (Fisher Scientific, Pittsburgh, PA, USA) and epicuticular wax was extracted by placing each individual sample into the chloroform and mildly agitating for 5 s. Afterwards, the organic solvent was evaporated with a nitrogen stream heated to 40 °C. After drying, 5 mL of 100 mg/L n-tetracosane (internal standard) in chloroform was added to reconstitute the extracted wax. The extract (0.3 mL per vial) was then transferred to Reacti-vials (Thermo Fisher Scientific) and subsequently evaporated under a gentle stream of nitrogen. The extract was then redissolved in a mixture of 150 µL bis-*N*,*N*-(trimethylsilyl) trifluoroacetamide (BSTFA) containing 1% trimethylchlorosilane (TMCS; Sigma-Aldrich, St. Louis, MO, USA) for derivatization. The vials were incubated at 75 °C for 70 min before the extract was injected into a gas chromatograph (Nexis GC-2030, Shimadzu, Japan) coupled to a GCMS (GCMS-QP 2020 NX, Shimadzu, Kyoto, Japan) for quantification. A capillary column (DB-5, Agilent, Santa Clara, CA, USA; 30 m, 0.25 mm, 0.25 m) was used for separation. Oven temperature was initially maintained at 150 °C for 1 min, then increased by 12 °C∙min^−1^ to reach 300 °C, which was maintained for 7 min. Both injector and detector temperatures were set to 270 °C. The flow rate of the helium carrier gas was 1.2 mL/min. The following mass spectrophotometry parameters were employed: inlet temperature, 250 °C; ion source temperature, 300 °C; and mass scan range was from 40 to 650.

Compound identification was based on NIST library and authentic standards including C7-C40 saturated alkanes standard mixture (Supelco, Bellefonte, PA, USA) and hexacosanol. Quantifications for some wax compounds were expressed as equivalent concentration using the standard alkanes (C30 for triterpenes) or hexacosanol (all alcohols).

### 2.10. Statistical Analysis

Experiments were performed in a completely randomized design. The data were analyzed by analysis of variance (ANOVA) using the Prism version 5.03 statistics software (GraphPad Software, San Diego, CA, USA), and significant differences were compared by one-way ANOVA following Tukey’s HSD tests for each experiment at *p* < 0.05. Pearson’s correlation analysis was conducted using MetaboAnalyst (https://www.metaboanalyst.ca).

## 3. Results and Discussion

### 3.1. O_2_ and CO_2_ Concentrations

**Figure 1:**
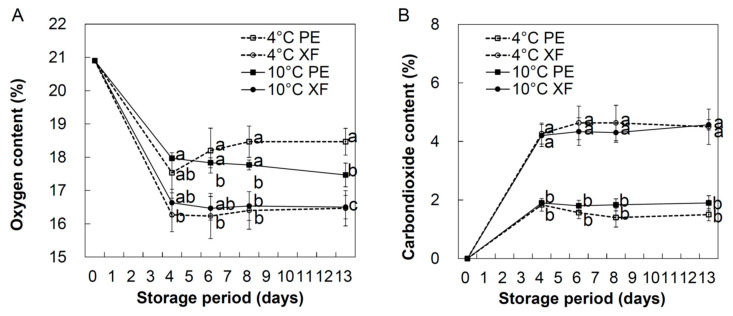
Oxygen (**A**) and carbon dioxide (**B**) concentration inside the “box-in-bag” packaging of oriental melons. The melons were packaged using polyethylene film (PE) or Xtend film (XF) and stored at 4 °C and 10 °C for 14 days. Data are presented as the mean ± SE of three replicates. Different letters indicate significant difference among treatments within the same storage temperature and storage time by Tukey’s HSD test with *p* < 0.05.

Oriental melons were harvested at optimal maturity and stored in a MAP of PE film or XF, at 4 °C or 10 °C, for 14 days. The initial atmosphere of both PE and XF packages was maintained throughout the experiments and contained ~20.9% O_2_ and ~0.1% CO_2_. During the cold storage period, O_2_ and CO_2_ concentration were relatively stable after four days of storage, regardless of the temperature. The O_2_ concentration in the PE film packages stored at 4 °C and 10 °C ranged between 17.5–18.5% and 17.4–17.9%, respectively, whereas O_2_ levels in the XF packages were lower (16.3–16.4% and 16.5–16.6%, respectively, Figure 1A). In contrast, XF showed a significantly higher CO_2_ concentration (4.2–4.6%) than that in PE (1.4–1.9%) packages, regardless of the temperature (Figure 1B). Overall, two MAP had significantly lower O_2_ and higher CO_2_ concentrations than that in unpackaging group (21% of O_2_ and 0.03% of CO_2_, data not shown here). In general, 3–8% CO_2_ and 2–5% O_2_ are recommended for MAP storage of fruits and vegetables [24]. Furthermore, a previous study suggested that the optimal controlled atmosphere for oriental melons was 2–3% O_2_ and 5–10% CO_2_ [2]. 

### 3.2. RH and Weight Loss

**Figure 2:**
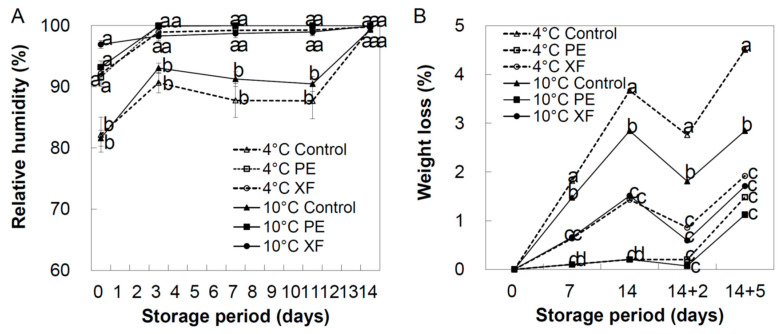
Relative humidity (**A**) and weight loss (**B**) inside the “box-in-bag” packaging of oriental melons. The melons were packaged using 0 polyethylene film (PE), Xtend film (XF), or no film treatment (control), and stored at 4 °C or 10 °C for 14 days. At day 14 + 2 the melons showed relatively lower weight loss compared with day 14, mostly due to the water condensing on the fruit and the box film, underestimating the actual weight loss of the fruit. Data are presented as mean ± SE of three replicates. Different letters indicate significant difference among treatments within the same storage temperature and storage time by Tukey’s HSD test with *p* < 0.05.

The RH within the PE packages was saturated under storage at both 4 °C and 10 °C within three days. In contrast, XF packages prevented water condensation inside and maintained high RH (~98%) during cold storage (Figure 2). The RH in the control was maintained at 87.2–93.0% from day 3 to day 11 of storage and increased up to 95% at day 14. Water condensation occurred inside PE packages; thus, no water loss occurred (Figure 2, RH changes in the two storage temperatures evaluated are available in Figure S1).

The fruit weight loss is presented in Figure 2. Control samples of oriental melons stored at 4 °C presented the highest loss in weight, with 3.67% and 4.5% of weight loss at day 14 and day 14 plus additional five days at retail display condition (14 + 5), respectively. However, PE and XF packaging treatment with refrigerated storage significantly reduced weight loss during the study period compared with the control samples. Minor weight loss was observed in samples packed in PE, irrespective of the storage condition at 4 °C or 10 °C (0.2% and 0.07%, respectively) during the 14 days. Although both PE and XF film packaging reach the same level of RH, weight loss rate (%) of the oriental melons was significantly lower in XF than in PE packaged samples during refrigerated storage at days 7 to 14. Permeability of the XF films to moisture and gases could be directly responsible to the weight loss. PE acted as a complete barrier to prevent moisture loss, whereas XF showed permeability to moisture at all storage temperatures, even at the retail display condition at 20 °C, because of its microperforation. These results indicated that weight loss is mainly a consequence of water content movement through the microperforation in the XF packaging, although water vapor was condensed on the PE packaging. Since PE acted as a barrier to water vapor release and helped maintain a high RH level, and consequently prevented weight loss of the fruits. It has been reported that MAP could extend the shelf life of fresh products by reducing their weight loss [25,26]. Nevertheless, when kept at 20 °C after 14 days of low temperature storage, the oriental melons showed significant weight loss, which could be attributed to higher respiration and transpiration rates at this marketing display temperature.

### 3.3. Fruit Quality: Firmness, Total Soluble Solids (TSS), and Surface Color

To evaluate the quality and shelf life of oriental melons, the fruits were stored at 4 °C or 10 °C for 14 days and then transferred to retail display conditions at 20 °C for another five days. Oriental melons stored in MAP at 10 °C for seven days showed the highest firmness, which decreased with storage time. Generally, fruits packed in XF or PE maintained their firmness better than their corresponding control fruits upon being transferred to 20 °C for five days (Table 1). In agreement with prolonged fruit quality, a previous study showed that MAP reduced the activity of enzymes involved in cell wall degradation [27].

For TSS content, significant differences among treatments at storage temperatures of 4 °C or 10 °C for 14 days were noticeable (Table 1). However, the trends of the different storing approaches were not consistent. Under retail display condition (20 °C) for two days, generally unpacked fruit showed higher TSS than MAP fruits, but the trend was not consistent throughout the storage duration. An increase in TSS content, particularly of sugars, may indicate ripening of the fruits, whereas the delay of this process could be due to the packaging process. An increase in TSS may also result from the breakdown of other complex sugars such as pectin, which is decomposed by the enzymes of the fruit.

The changes in fruit surface color over the storage period were measured as Hue value from both suture (Table 1) and peel (Figure S2G,H). The Hue value change of the suture was more obvious than that of the peel. Thus, Table 1 only shows Hue value of peel, whereas suture of Hue value changes is presented in Figure S2. Surface color evaluations showed significant Hue value differences in white sutures on fruits between 4 °C and 10 °C stored oriental melon at days 14 + 2 and 14 + 5. The unpacked fruit control showed lower Hue value than both MAP fruits at retail display condition (20 °C) after 14 days of cold storage. The suture of control had slightly yellowing showing lower hue value (88.3) compared to PE and XF packaged fruit, 92.0 and 90.8, respectively at 14 + 5 days. Similar report showed apples stored in MA packs presented better color than fruits stored in air showing the higher L* and hue values and lower a*value after 6 month cold storage [28]. Lightness (L*) and green to red (a*) from Hunter’s L*a*b* values were mostly significantly different in both peel or suture between treatment conditions, at either 4 °C or 10 °C storage. Lightness gradually decreased during storage time, but MAP treatments significantly inhibit lightness reduction of the peel or suture. This effect of MAP was more obvious in samples stored at 4 °C than in those stored at 10 °C. Decreasing lightness of the peel and suture at 4 °C maybe related with CI, similar to browning. Taken together, storage temperature and MAP have significant impact on skin and suture color of the oriental melons (Figures S2 and S3, Table 1). These results indicated that modified the atmospheric condition, and the high humidity inside the packages slowed down the ripening and softening processes. However, previous reports described that XF10 liners had no significant effects on other fruit quality parameters, including decay, juice TSS and acid content, and citrus fruit taste [19].

### 3.4. Browning of the Fruit Suture and Tissue Structure

**Figure 3:**
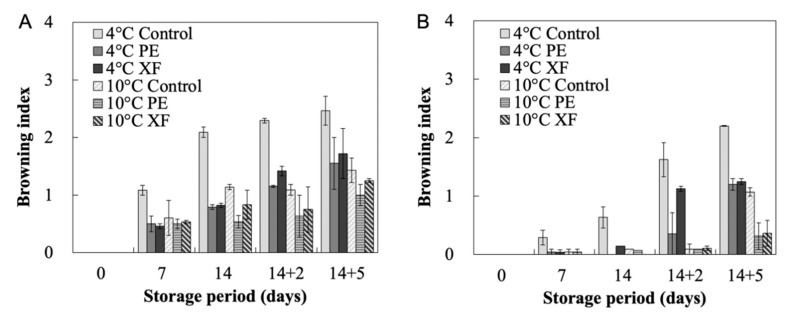
Effect of modified atmosphere packaging on peel browning symptom of white linear sutures (**A**) and peel (**B**) of oriental melons. The fruits were packaged in a commercial box made of polyethylene (PE) or Xtend film (XF) and stored at 4 °C or 10 °C for 14 days (14). Afterwards, the melons were transferred to 20 °C condition for five days (14 + 5). Fruits stored in a standard commercial box were used as controls.

In our experiments, browning was observed in the control fruit during cold storage. In contrast, only one or two of the 15 MAP treated fruits showed less than 5% of fruit surface browning during 14 days of cold storage. Browning increased after 14 days at 4 °C and 10 °C followed by five days of storage at 20 °C (Figure 3); however, MAP with either PE or XF packaging considerably reduced peel and sutures browning compared with control samples. Storage temperature also affected oriental melon browning process. Notably, fruits stored at 4 °C showed severe peel and white liner suture browning compared with fruits stored at 10 °C. Consistent with these results, lower temperatures have been found to induce browning in muskmelons [8].

**Figure 4:**
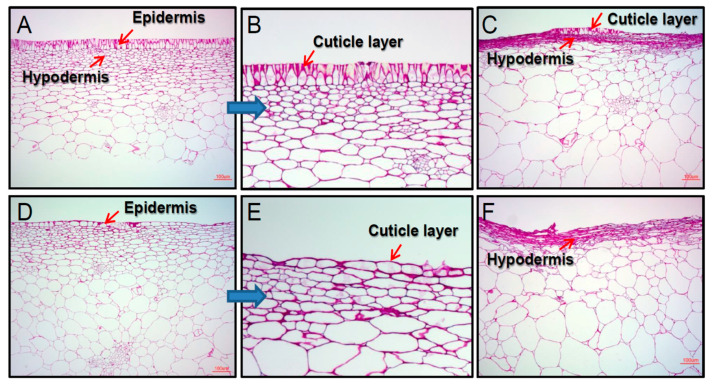
Anatomical analysis of oriental melon peel and suture. Light microscope images of yellow peel tissue: (**A**), normal tissue; (**B**), amplified of image (**A**); and (**C**), browning symptom tissue. Light microscope images of white linear suture tissue: (**D**), normal tissue; (**E**), amplified of image (**D**); and (**F**), browning symptom tissue.

In addition, browning symptom of the suture was more severe than browning of the fruit peel tissue. For the same storage period, the incidence of suture browning was up to 5–10 times higher than fruit yellow peel. Fruit browning is the main contributor to the postharvest loss of oriental melons. The white linear sutures (2.25 ± 0.56 μm, *n* = 26) of oriental melons, unlike the peels (19.98 ± 6.00 μm, *n* = 26), have an epidermis layer with much less cuticular cells (Figure 4A,B,D,E). In other words, the peel had an 8.88 times ticker cuticle layer. Cross section of both browning damaged fruit peel and suture tissues showed very compact cell size and shrinking cell morphology (Figure 4C,F), suggesting severe water loss in the hypodermis layer. Interestingly, even in the browning area on the fruit peel surface, epidermis cells were not substantially shirked compared with the hypodermis layer (Figure 4C). This result suggests that well-developed cuticle layer on the surface effectively prevents water loss. The browning of oriental melon peels and white linear sutures may have been caused by cell membrane impairment in the hypodermis layer, which suffered water loss during the long-term low temperature storage (Figure 4). Disruption of the cell membrane integrity could have caused lipid peroxidation by exposing cell membrane lipids to more O_2_. Even if a similar water loss has occurred in the same suture tissue in both 4 °C and 10 °C stored samples, lower temperature stored fruit showed more severe browning symptom at 4 °C. This may be caused by the imbalance of the antioxidant system of the fruit [29]. Fruit cuticle is the outer physical barrier that protects it from external stresses and helps maintain its internal structure and water content. A recent review paper on fruit cuticles reported a strong relationship between the cuticle features and susceptibility to fungal diseases [30].

### 3.5. Epicuticular Wax and Specific Water Loss in Oriental Melon Sutures

**Figure 5:**
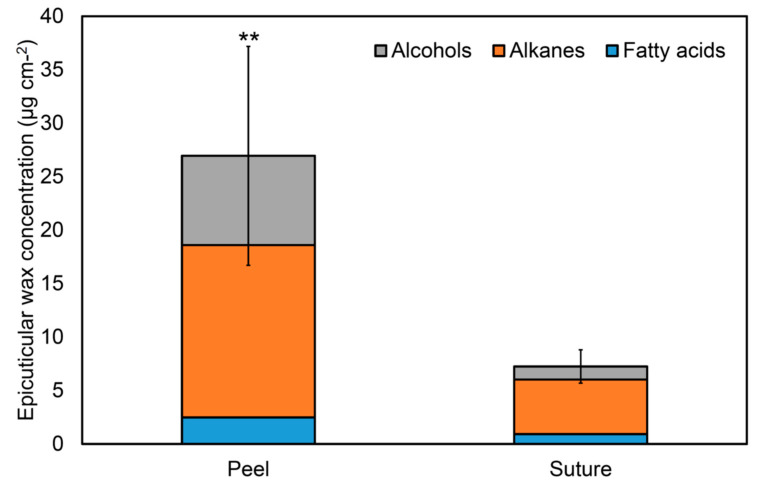
Comparison of epicuticular wax on oriental melon peel and sutures. Data are presented as mean ± SD of three replicates. Asterisks (**) indicate significant difference of total epicuticular wax between peel and suture by Student’s t-test with *p* < 0.01.

The water loss in the white linear suture area was particularly marked, which could be due to the reduced thickness of the cuticle layer. Moreover, it may relate with the epicuticular wax difference on the fruit surface. The yellow peel tissue of the oriental melon has a completely different texture feeling as compared with the sutures surface, with the yellow peel surface being oily and greasy, whereas the white suture surface has a non-greasy feeling. As shown in Figure 5, long chain alkanes, long chain alcohols and fatty acids were identified as the major epicuticular wax component on oriental melon surface. Total wax concentration of yellow fruit peel was significantly higher than that of white suture (26.95 vs. 7.25 µg cm^−2^). Long chain alkanes were accounted for 59.8% and 70.2% of total wax components on yellow peel and white suture, respectively. Among them, hentriacontane (C_31_ alkane) was the major alkane of total waxes on both yellow peel and white suture (21.8 and 21.6% of total wax components, respectively), followed by nonacosan (C_29_ alkane; 14.8% and 18.9% of total wax components, respectively). In yellow peel, long chain alcohols were account for 31% of total wax components, and included octacosanol (C_28_ alcohol), heptacosanol (C_27_ alcohol), and hexacosanol (C_26_ alcohol) as the major alcohols (8.6%, 7.1%, and 6.9% of total wax components, respectively). In white suture, long chain alcohols account for 17% of total wax components, and included docosanol (C_22_ alcohol), octacosanol (C_28_ alcohol), and tetracosanol (C_24_ alcohol) as major alcohols (3.4%, 3.2%, and 2.9% of total wax components). Fatty acids were account for 9.2% and 12.8% of total wax components on yellow peel and white suture, respectively. Oleic acid was the major fatty acid on yellow peel (4.8% of total wax components), whereas stearic acid was the major fatty acid on white suture (7.4% of total wax components). This result was consistent with a recent study on smooth surface melon, such as honeydew [31]. A correlation between the epicuticular wax and water content loss were reported for several fruits, including mulberries and peppers [32,33]. In blueberries, the organellar membrane structure was disrupted upon cuticular wax removal [34]. Chu et al. [34] also reported that wax removal decreased the activities of antioxidant enzymes and the antioxidant content of peppers, and accelerated accumulation of reactive oxygen species (ROS) and lipid peroxidation, especially at the later period of storage. In addition, epicuticular wax crystals can change hydrophobicity of a plant surface and its susceptibility of food pathogen [35,36]. In this study, the difference in epicuticular waxes between yellow peel and white suture was found out. These results suggest that differential susceptibility to browning on oriental melon surface by area was due to the difference in epicuticular waxes. In addition to major wax components, unknown triterpenes were also detected (Figure S4 and Table S2); they were significantly higher on yellow peel surface (25.47 μg cm^−2^) than on white suture (4.03 μg cm^−2^). In plant belonging to the Cucurbitaceae family, cucurbitacins are known as triterpenes [37,38]. Although the unknown triterpenes showed 93% similarity to glutinol from NIST library, further studies are needed to examine the identification and role of triterpenes presented on oriental melon surface for physiological change at postharvest.

**Figure 6:**
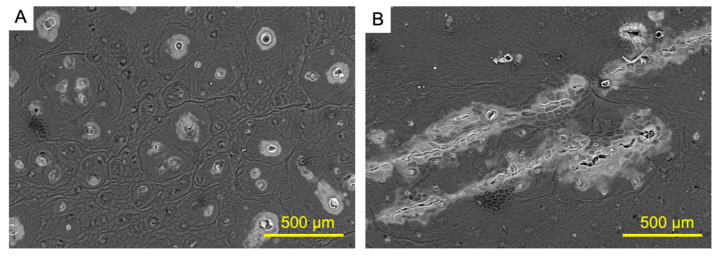
Scanning electron microscope images from normal oriental melon fruit suture surface (**A**) and brown surface of fruit suture (**B**).

To visualize the trace of weight loss from fruit suture, the surface image of oriental melon was taken using scanning electron microscope. Intact suture surface did not show any microcrack (Figure 6A) while browning area on suture surface showed microcrack (Figure 6B). The observed microcracks were probably occurred by water loss for its the lower levels of cuticle layer thickness and epicuticular wax deposit. These results also confirm that browning of suture surface was accelerated as dehydration.

### 3.6. Marketability Change by Modified Atmosphere/Modified Humidity Packeging (MAP/MHP)

**Figure 7:**
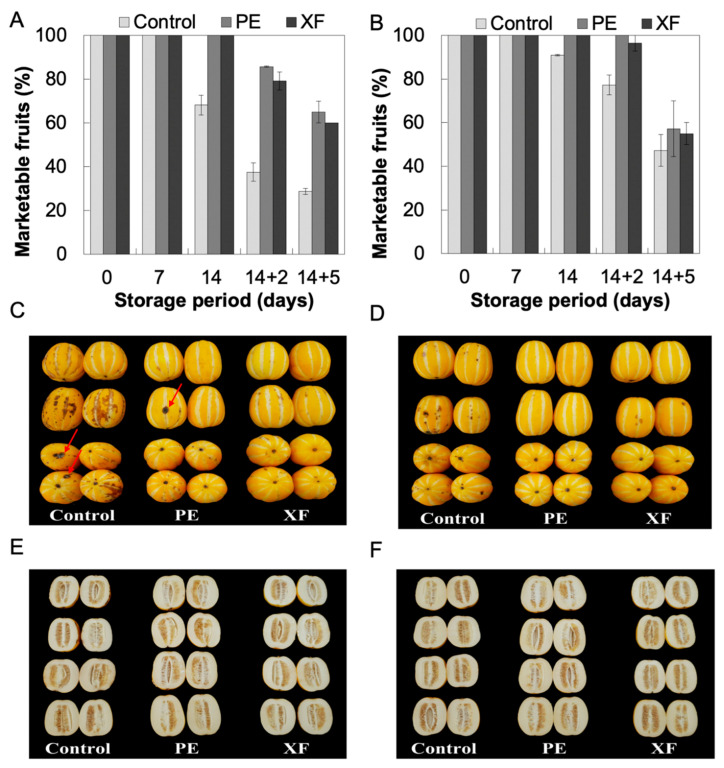
Effect of modified atmosphere packaging on overall appearance of oriental melons. Marketability of oriental melons that were stored at 4 °C (**A**) or 10 °C (**B**) for 14 days (14) and transferred to 20 °C for another two or five days (14 + 2 or 14 + 5). Representative images from oriental melon stored at 4 °C (**C**,**E**) or 10 °C (**D**,**F**) for 14 days, and transferred to 20 °C for another five days. Red arrows in C indicate mold damage by fungi.

An evaluation of the percentage of marketable fruit, upon transfer to 20 °C for two days after 14 days of refrigerated storage (4 °C), showed that unpackaged controls started with 37.5% of marketable fruit, whereas in PE or XF packed fruit, the initial percentage of marketable fruits were 85.7% and 79.1%, respectively (Figure 7A). Fruit analysis at day 14 + 5 also showed that PE (65%) and XF (60%) packaging achieved more than twice marketable fruit compared with unpacked fruit (28.6%). The marketability of unpackaged control fruits dramatically decreased under retail display conditions after cold storage compared with PE and XF packed fruits. However, there was no notable difference of marketability between PE and XF packed fruits stored under the same conditions for 14 + 2 and 14 + 5 days. However, the melons decayed more frequently within the PE-treated group than in the XF-treated group (Figure 7C,D), with about 25% more decayed fruit being observed in PE than XF. Unfortunately, we did not evaluate decay incidence from the treatments this study. It is possible that excessive humidity in the PE could promote decay of the fruit. Thus, MAP/MHP in XF packages provides an advantage for fruits that are sensitive to excess condensed water inside the package. According to our previous study [21], tomatoes treated with XF packaging showed numerically lower decay rate than with PE packaging; however, the differences were not statistically significant. 

To store oriental melons for long periods or transporting them long distances, low temperature storage is necessary to reduce their metabolism, including respiration and ethylene production, and thus maintain its freshness [39]. However, oriental melons stored at low temperatures (3–7 °C) are susceptible to CI. Overall, fruits stored at 10 °C showed better marketability than that stored at 4 °C, since oriental melon fruits at 4 °C storage showed lower CI, such as browning (Figure 7C,D), which consequently leads to poor overall appearance and low marketability. Moreover, below the browning tissue symptom, the fruits showed brown tissue color with compromised firmness upon 4 °C and 10 °C control (Figure 7E,F). These results showed that MAP reduced browning symptom on peel and white linear suture and improved oriental melon marketability. In agreement, prior results showed that MAP prevents CI symptoms in tomatoes [21] and XF packaging reduced CI development in oranges after six weeks of cold storage at 2 °C and five days under retail display conditions [19].

Parat et al. [19] mentioned some potential disadvantages of MAP. For example, MAP may enhance anaerobic respiration and the development of off-flavor, and excessive humidity may increase decay incidence. Parat et al. [19] also reported that different perforation size in XF films could significantly affect the gas composition in the “bag-in-box”, implying that is still possible to optimize microperforation for oriental melon to achieve an optimized gas/humidity atmosphere. MAP can be used for both packaging and storage purposes with low cost. Thus, MAP could be helpful to ameliorate CI of oriental melon during cold storage for long distance transportation.

### 3.7. Correlation between Modified Atmospherepakaging (MAP) and Cold Injuries (CI)

**Figure 8:**
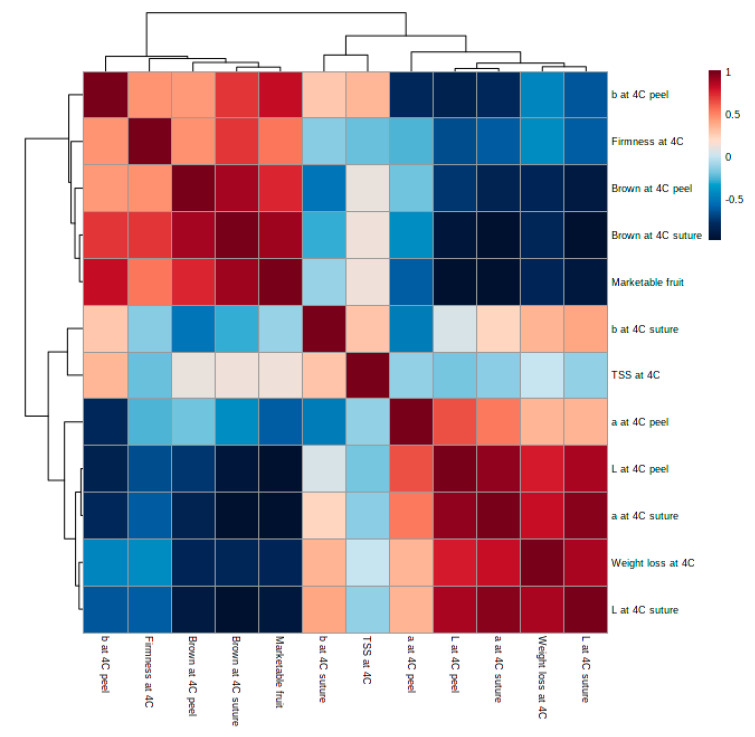
Correlation analysis between various fruit quality parameters during refrigerated storage (4 °C) and transferred to 20 °C for additional two or five days.

To determine the CI reducing effect of MAP on oriental melon stored at 4 °C, correlation analyses were conducted using brown index to quantify CI. Significant correlations are listed in Table S1 and also presented in Figure 8. Browning was mostly observed on white sutures of the fruit, and was found to be strongly correlated with Hunter’s a* value at 4 °C suture (r = −0.969, *p* < 0.001, *n* = 15), as well as with the L* value at 4 °C suture (r = −0.961, *p* < 0.001, *n* = 15). Browning of suture at 4 °C was also strongly correlated with the L* value at 4 °C peel (r = −0.961, *p* < 0.001, *n* = 15). The marketable fruit percentage was strongly correlated with the L* value at 4 °C peel (r = −0.965, *p* < 0.001, *n* = 15), as well as with the a* value at 4 °C suture (r = −0.963, *p* < 0.001, *n* = 15). Visual color is one of the most important visual attributes to consumers. When browning symptom visually shows on the surface of oriental melon fruit the lightness is dramatically reduced (Figure 7C,D).

**Figure 9:**
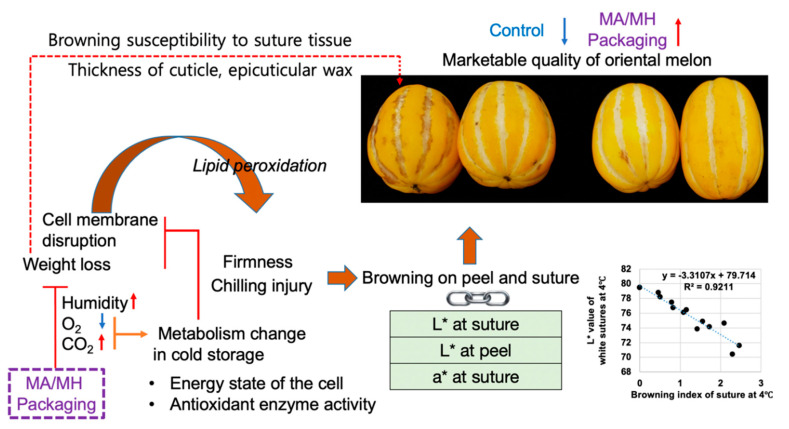
Modified atmosphere packaging (MAP)-mediated cold injury preventing effect on oriental melon during refrigerated (4 °C) storage. L* and a* indicate lightness and degree of redness from red to green, respectively.

Altogether, the L* value changes on the peel and suture of oriental melon may be related with the browning process by CI (Figure 9). To date, no quantitative method is available to measure accurately the browning of oriental melon induced by cold storage. To the best of our knowledge, this study is the first to identify quantitative parameters of measuring CI-related browning of oriental melon. MAP decreased O_2_ and increased CO_2_ and humidity inside the packaging, resulting in reduced metabolism, weight loss, and CI degree. The water loss on the peel tissue may have a negative influence on the membrane structure and disruption of cellular compartmentalization [40]. A previous study on kiwifruit reported that gradual cooling had higher superoxide dismutase, catalase, ascorbate peroxidase, and peroxidase activities than the fruit treated by direct cooling during storage [29]. Based on this report, higher antioxidant activity may contribute to reduced CI symptoms. In our study, we directly store oriental melon at 4 °C, which may have attributed to lower antioxidant activity. Moreover, during the cold storage of oriental melon, ROS production might have been promoted by the impaired energy state of the cells and/or could have contributed for disruption of cell membrane integrity [41]. Indeed, membrane lipid peroxidation may be one of the first event of cold injury [41], and lipid peroxidation was reported as a CI symptom [29,39]. Our previous report showed that optimal package atmosphere conditions in MAP could lead to increased antioxidant levels, which in turn could improve the freshness of tomatoes by reducing CI during cold storage, even at retail display conditions [21]. In the present study, thicker cuticle layer with higher epicuticular wax deposits on the yellow peel of oriental melon can possibly explain why the peel tissue is less susceptible to browning than the white linear suture during cold storage. The higher water loss on the white linear suture tissue may also lead to cell membrane disruption and lipid peroxidation, ultimately resulting in browning symptom during cold storage. In addition, the yellow peel tissue of oriental melon contains antioxidant carotenoids, including lutein and β-carotene [42], which may have an important role on reducing lipid peroxidation. It has been reported that grapefruits with high accumulation of lycopene were highly resistant to CI upon subsequent postharvest cold storage [43]; however, mechanistic details that could explain this observation still remain to be elucidated.

## 4. Conclusions

Preventing CI is critical for extending the shelf life and maintaining the postharvest quality of oriental melons during storage, transport, and retailing. This study provided experimental data that revealed that suture specific browning, as a CI symptom, is associated with impaired cuticle layer and reduced epicuticular wax protection. This study also showed that MAP with PE or XF can effectively prevent browning symptoms and prolong the freshness of oriental melon by using a modified storage environment with elevated CO_2_ (1.8–4.6%) and reduced O_2_ contents (16.2–18.5%) during cold storage. Moreover, optimal RH adjusted by MAP may influence the oriental melon quality, demonstrating to be ideal for its storage by minimizing weight loss and maintaining firmness. In contrast, MAP had little influence on TSS. Furthermore, MAP effectively reduced the browning index and improved melon marketability compared with standard, unpackaged conditions. Altogether, this study showed that the use of PE and XF packing materials can modify the packaging atmospheric conditions to maintain the quality of oriental melons during cold storage and retail display conditions.

## Figures and Tables

**Table 1:** Firmness, soluble solid content, and Hue under modified atmosphere film of oriental melon during 4 °C and 10 °C storage.

Storage Time (Days)	Storage Temperature (°C)	Treatment	Firmness (N)	TSS	Hue of Suture
0	4	Control	19.45 ± 0.62 ^a^	11.60 ± 0.10 ^a^	96.70 ± 0.33 ^a^
		PE	19.45 ± 0.62 ^a^	11.60 ± 0.10 ^a^	96.70 ± 0.33 ^a^
		XF	19.45 ± 0.62 ^a^	11.60 ± 0.10 ^a^	96.70 ± 0.33 ^a^
0	10	Control	19.45 ± 0.62 ^a^	11.60 ± 0.10 ^a^	96.70 ± 0.33 ^a^
		PE	19.45 ± 0.62 ^a^	11.60 ± 0.10 ^a^	96.70 ± 0.33 ^a^
		XF	19.45 ± 0.62 ^a^	11.60 ± 0.10 ^a^	96.70 ± 0.33 ^a^
7	4	Control	14.01 ± 0.45 ^a^	12.17 ± 0.09 ^b,c,d^	94.77 ± 0.36 ^a^
		PE	18.54 ± 0.37 ^b,c^	12.27 ± 0.13 ^d,e^	95.57 ± 0.40 ^a^
		XF	16.57 ± 0.56 ^b^	12.77 ± 0.03 ^e^	95.80 ± 0.32 ^a^
7	10	Control	18.93 ± 0.37 ^c^	11.60 ± 0.15 ^a^	94.92 ± 0.55 ^a^
		PE	19.26 ± 0.34 ^c^	11.70 ± 0.15 ^a,bc^	95.62 ± 0.46 ^a^
		XF	19.86 ± 0.82 ^c^	11.70 ± 0.07 ^ab^	95.79 ± 0.34 ^a^
14	4	Control	19.52 ± 0.41 ^b^	11.60 ± 0.06 ^a,b^	91.35 ± 0.75 ^a^
		PE	18.06 ± 0.74 ^a,b^	11.27 ± 0.09 ^a^	95.60 ± 0.52 ^b^
		XF	19.09 ± 0.45 ^b^	11.73 ± 0.12 ^a,b,c^	94.35 ± 0.42 ^b^
14	10	Control	17.67 ± 0.50 ^a,b^	12.20 ± 0.03 ^c^	91.66 ± 1.04 ^a^
		PE	16.61 ± 0.52 ^a^	11.70 ± 0.17 ^a,b,c^	93.60 ± 0.36 ^a,b^
		XF	17.82 ± 0.33 ^a,b^	12.00 ± 0.09 ^b,c^	94.76 ± 0.42 ^b^
14 + 2	4	Control	14.00 ± 0.46 ^a^	12.03 ± 0.09 ^b^	88.22 ± 0.62 ^a^
		PE	16.74 ± 0.44 ^a,b,c,d^	12.17 ± 0.03 ^b^	92.71 ± 0.66 ^b^
		XF	14.46 ± 0.59 ^a,b^	11.23 ± 0.13 ^a^	91.90 ± 0.83 ^b^
14 + 2	10	Control	15.00 ± 0.37 ^a,b,c^	13.30 ± 0.06 ^c^	92.70 ± 0.79 ^b^
		PE	17.93 ± 0.43 ^d^	11.00 ± 0.06 ^a^	93.77 ± 0.46 ^b^
		XF	17.01 ± 0.69 ^c,d^	12.50 ± 0.13 ^b^	92.88 ± 0.60 ^b^
14 + 5	4	Control	14.86 ± 0.65 ^a^	12.00 ± 0.40 ^b^	88.32 ± 1.13 ^a^
		PE	16.74 ± 0.31 ^a,b^	11.27 ± 0.15 ^a,b^	92.06 ± 0.56 ^b,c^
		XF	16.35 ± 0.55 ^a,b^	11.07 ± 0.18 ^a^	90.83 ± 0.88 ^ab^
14 + 5	10	Control	16.22 ± 0.32 ^a^	11.20 ± 0.13 ^a,b^	91.98 ± 0.70 ^b,c^
		PE	18.22 ± 0.53 ^b^	10.40 ± 0.03 ^a^	94.69 ± 0.38 ^c^
		XF	15.05 ± 0.46 ^a^	12.00 ± 0.09 ^b^	94.40 ± 0.37 ^c^

Control, non-film treatment; PE film, 0.03 mm polyethylene; X-tend Film, manufactured from blends of polyamides with other polymeric and non-polymeric compounds. Data represents the means ± standard deviation (*n* = 30). Different letters indicate significant difference among treatments within the same storage temperature and storage time by Tukey’s HSD test with *p* < 0.05.

## Supplementary Materials

The following are available online at https://www.mdpi.com/2304-8158/9/9/1329/s1, Figure S1: Temperature and relative humidity inside the “box-in-bag” of oriental melon. Figure S2: Hunter’s L a b value and Hue value for visual color changes of oriental melon peel stored in different packaging and temperatures. Figure S3: Hunter’s L*a*b* value and Hue value for visual color changes of oriental melon sutures stored in different packaging and temperatures. Figure S4: Gas chromatograph–mass spectrometry (GC-MS) chromatogram of expicuticular wax analysis. Table S1: Significant Pearson’s correlation analysis between fruit quality indices. Table S2: Epicuticular wax concentration of fruit surface (peel and suture).
