# Supplementary material for: Modified Atmosphere and Humidity Film Reduces Browning Susceptibility of Oriental Melon Suture Tissue during Cold Storage
Source: Foods. 2020 Sep 21;9(9):1329. doi: 10.3390/foods9091329 (PMC7555987; doi:10.3390/foods9091329)

foods

Type of the Paper (Article)

Modified atmosphere and humidity film reduces browning susceptibility of oriental melon suture tissue during cold storage

Me-Hea Park[1]*, Eun-Ha Chang[1], Hae- Jo Yang[1], Jung-Soo Lee[1], Gyung-Ran Do[2] Hyun Jong Song[3], Min-Sun Chang[1] and Kang-Mo Ku[3,*]

[1] Postharvest Research Division, National Institute of Horticultural & Herbal Science, Wanju, 55365, Republic of Korea; poemmich@korea.kr
[2] Planning and Coordination Division, National Institute of Horticultural & Herbal Science, Wanju, 55365, Republic of Korea;microdo@korea.kr
[3] Department of Horticulture, College of Agriculture and Life Sciences, Chonnam National University, Gwangju, 61186, Republic of Korea; ku9@jnu.ac.kr
* Correspondence: KKM – ku9@jnu.ac.kr, Tel. +82-062-530-2065; MHP – poemmich@korea.kr, Tel. +82-063-238-6512

Figure S1. Temperature and relative humidity inside the "box-in-bag." experiment of oriental melon.

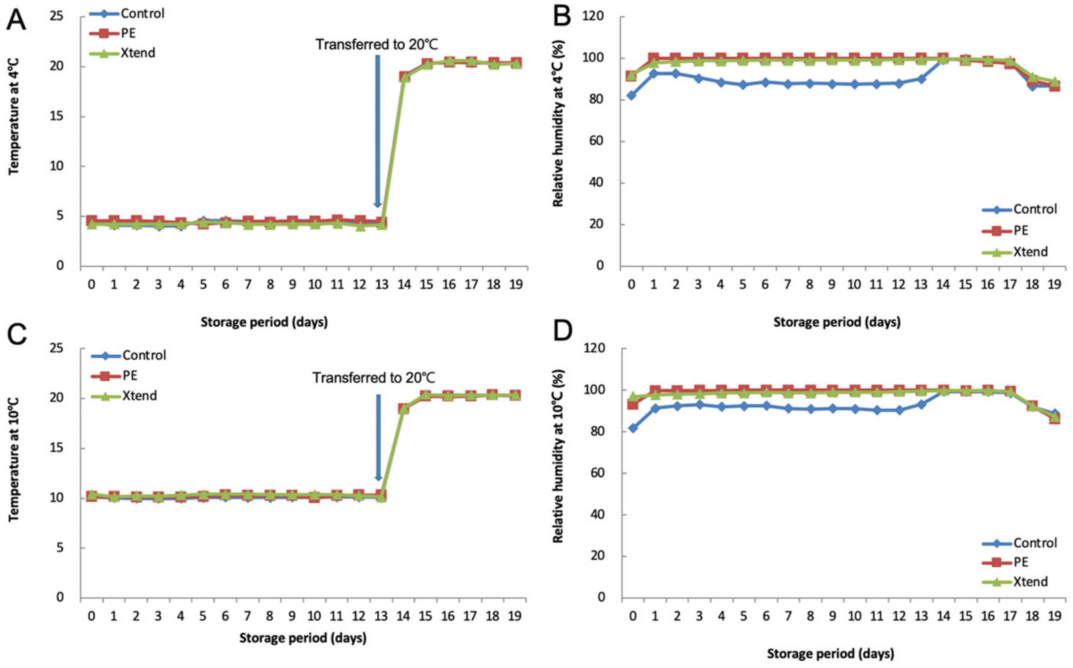

Figure S2. Hunter's L* a* b* value and Hue value for visual color changes of oriental melon peel stored in different packaging at different storage temperatures.

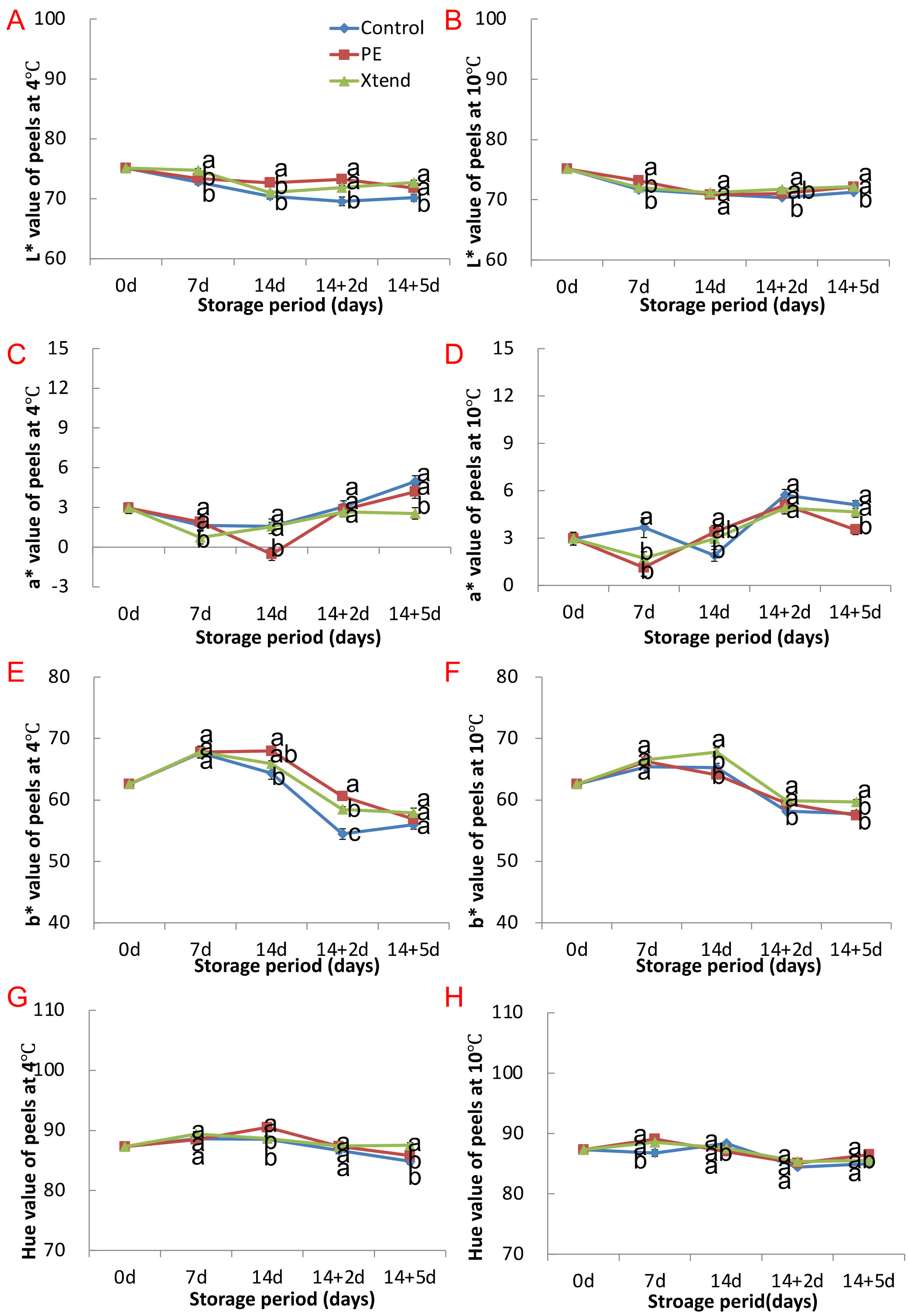

Figure S3. Hunter's L* a* b* value and Hue value for visual color changes of oriental melon sutures stored in different packaging at different storage temperatures.

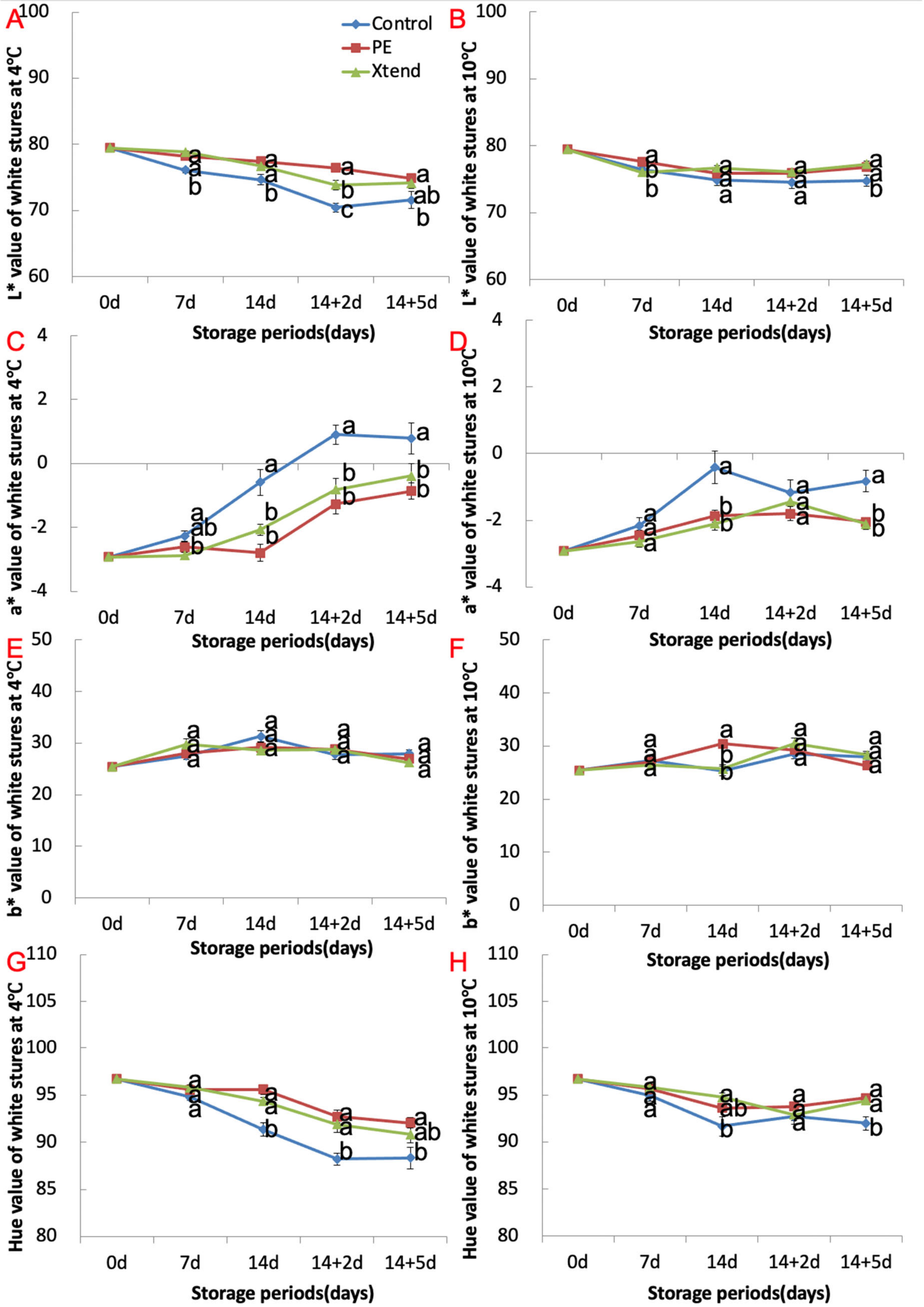

Table S1. Significant Pearson's correlation analysis between fruit quality indices

Feature1	Feature2	Correlation [pearson]	P.value
a* at 4C suture	Brown at 4C suture	-0.968765806	2.99E-09
L at 4C peel	Marketable fruit	-0.964561229	6.73E-09
a* at 4C suture	Marketable fruit	-0.962532145	9.62E-09
L* at 4C suture	Brown at 4C suture	-0.961316167	1.18E-08
L* at 4C suture	a* at 4C suture	0.958132567	1.96E-08
L* at 4C peel	a* at 4C suture	0.945303917	1.08E-07
L* at 4C peel	Brown at 4C suture	-0.924438445	8.37E-07
Brown at 4C suture	Marketable fruit	0.912031578	2.18E-06
L* at 4C suture	Marketable fruit	-0.908630347	2.77E-06
L* at 4C suture	Brown at 4C peel	-0.898217264	5.44E-06
Brown at 4C suture	Brown at 4C peel	0.894617937	6.76E-06
L* at 4C suture	L* at 4C peel	0.889226295	9.22E-06
Weight loss at 4C	L* at 4C suture	0.885048311	1.16E-05
L* at 4C peel	b* at 4C peel	-0.839563042	9.04E-05
a* at 4C suture	Brown at 4C peel	-0.837490773	9.78E-05
b* at 4C peel	Marketable fruit	0.826356799	0.000146183
Weight loss at 4C	Marketable fruit	-0.820695717	0.000177485
Weight loss at 4C	Brown at 4C peel	-0.8162778	0.000205565
Weight loss at 4C	a* at 4C suture	0.815742012	0.000209205
Weight loss at 4C	Brown at 4C suture	-0.808828167	0.000261113
a* at 4C peel	b* at 4C peel	-0.804032957	0.000302963
a* at 4C suture	b* at 4C peel	-0.801074369	0.000331413
Weight loss at 4C	L* at 4C peel	0.780744586	0.00059174
Brown at 4C peel	Marketable fruit	0.748147985	0.001337285
L* at 4C peel	Brown at 4C peel	-0.733308996	0.001865255
b* at 4C peel	Brown at 4C suture	0.711935089	0.002907742
Brown at 4C suture	Firmness at 4C	0.709025722	0.003079839

L* at 4C peel	Firmness at 4C	-0.655415853	0.007990155
L* at 4C peel	a* at 4C peel	0.642349643	0.009816462
L* at 4C suture	b* at 4C peel	-0.627271953	0.012312248
a* at 4C suture	Firmness at 4C	-0.608438167	0.016091869
a* at 4C peel	Marketable fruit	-0.606935341	0.016428046
L* at 4C suture	Firmness at 4C	-0.605510801	0.016751681
Marketable fruit	Firmness at 4C	0.541188194	0.037217094
a* at 4C suture	a* at 4C peel	0.536450109	0.03924483

Table S2. Epicuticular wax concentration of fruit surface (peel and suture)

**Wax composition**	**Peel ($\mu g\ cm^{-2}$)**	**Suture ($\mu g\ cm^{-2}$)**
palmitic acid	0.36±0.16	0.27±0.04
oleic acid	1.30±0.71	0.13±0.01
stearic acid	0.82±35	0.54±0.09
tricostan (C23)	0.62±0.19	0.08±0.01
pentacosan (C25)	0.33±0.09	0.09±0.01
hexacosan (C26)	0.11±0.04	0.04±0.01
heptacosan (C27)	0.91±0.22	0.34±0.04
octacosan (C28)	0.36±0.14	0.15±0.02
nonacosan (C29)	3.99±0.5	1.37±0.2
triacontane (C30)	0.76±0.21	0.28±0.06
hentriacontane (C31)	5.87±0.65	1.89±0.28
dotriacontane (C32)	0.74±0.2	0.25±0.03
tritriacontane (C33)	1.09±0.16	0.39±0.06
tetratriacontane (C34)	0.38±0.06	0.09±0.03
pentatriacontane (C35)	0.97±0.14	0.11±0.03
docosanol (C22)	0.50±0.09	0.25±0.04
tricosanol (C23)	0.33±0.12	0.08±0.01

tetracosanol (C24)	0.85±0.26	0.21±0.04
pentacosanol (C25)	0.58±0.21	0.11±0.01
hexacosanol (C26)	1.85±0.71	0.17±0.04
heptacosanol (C27)	1.92±0.71	0.19±0.04
octacosanol (C28)	2.32±0.82	0.23±0.07
**Total waxes**	**26.95±6.44**	**7.25±0.95**
Unknown triterpene1 (Glutinol?)	20.81±4.44	2.99±0.64
Unknown triterpene2 (Glutinol?)	3.16±0.79	0.53±0.1
Unknown triterpene3 (Glutinol?)	1.50±0.39	0.51±0.16
**Total triterpenes**	**25.47±5.33**	**4.03±0.89**

Figure S4. Gas chromatograph–mass spectrometry (GC-MS) chromatogram of expicuticular wax analysis.

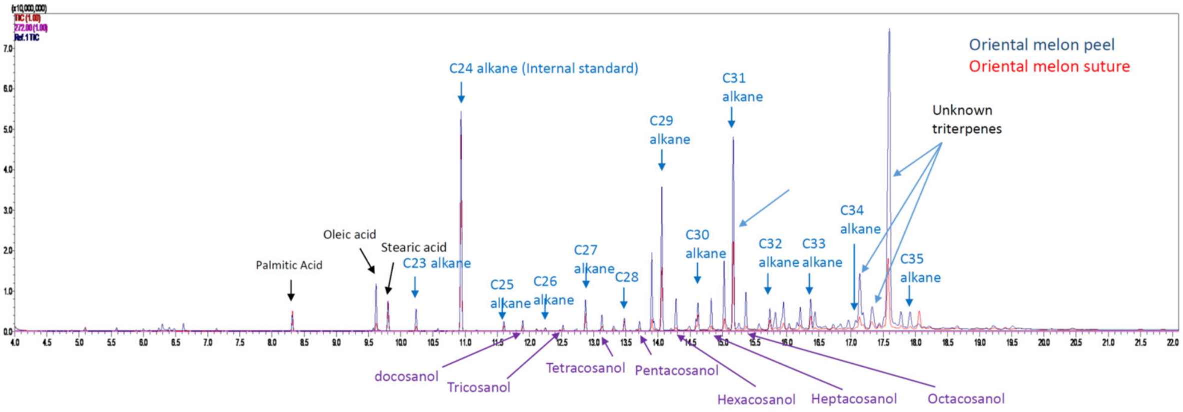

Supplement: Supplementary file 1 [file foods-09-01329-s001.pdf]
